# Efficacy, safety, and biomarkers of neoadjuvant trastuzumab and pertuzumab combined with chemotherapy in Chinese patients with HER2-positive breast cancer: a multicenter retrospective cohort study

**DOI:** 10.1097/JS9.0000000000003551

**Published:** 2025-09-30

**Authors:** Xiaowei Qi, Hong Hu, Pengfei Qiu, Wenlin Chen, Xiaochun Wang, Qiyun Shi, Yan Xu, Shu Liu, Yanman Fang, Taolang Li, Jia Ming, Sihai Zhou, Fan Chai, Yueyang Liang, Yuanming Fan, Peng Tang, Li Chen, Shushu Wang, Jun Jiang, Mengyuan Wang, Yi Zhang, Jianyun Nie, Yongsheng Wang

**Affiliations:** a Department of Breast and Thyroid Surgery, Southwest Hospital, Army Medical University, Chongqing, China; b Department of Breast and Thyroid Surgery, Shenzhen People’s Hospital, Shenzhen, Guangdong, China; c Breast Cancer Center, Shandong Cancer Hospital Affiliated to Shandong University, Jinan, Shandong, China; d Third Department of Breast Surgery, Peking University Cancer Hospital Yunan, Yunnan Cancer Hospital, The Third Affiliated Hospital of Kunming Medical University, Kunming, Yunnan, China; e Department of Breast Surgery, Affiliated Hospital of Hebei University, Baoding, Hebei, China; f Department of thoracic surgery, The Eighth Medical Center of Chinese PLA General Hospital, Beijing, China; g Department of Breast and Thyroid Surgery, Army Medical Center of PLA, Chongqing, China; h Breast Cancer Center, Affiliated Hospital of Guizhou Medical University, Guiyang, Guizhou, China; i Breast Specialty, Guiyang Maternal and Child Health Care Hospital, Guiyang, Guizhou, China; j Department of Breast and Thyroid Surgery, Affiliated Hospital of Zunyi Medical University, Zunyi, Guizhou, China; k Department of Breast and Thyroid Surgery, The Second Affiliated Hospital of Chongqing Medical University, Chongqing, China; l Department of General Surgery, Yongchuan Hospital Affiliated of Chongqing Medical University, Chongqing, China; m Department of Breast and Thyroid Surgery, Chongqing People’s Hospital, Chongqing, China; n Department of Breast and Thyroid Surgery, The Third Affiliated Hospital of Chongqing Medical University, Chongqing, China; o Department of General Surgery, Chongqing Changshou District People’s Hospital, Chongqing, China; p Breast Cancer Center, Chongqing Three Gorges Central Hospital, Chongqing, China

**Keywords:** HER2-positive breast cancer, neoadjuvant chemotherapy, pathological complete response, pertuzumab, trastuzumab

## Abstract

**Objective:**

This multicenter real-world study aimed to evaluate the efficacy, safety, and neoadjuvant trastuzumab and pertuzumab combined with different chemotherapy regimens in Chinese patients with human epidermal growth factor receptor 2 (HER2)-positive breast cancer.

**Methods:**

A retrospective analysis was conducted on 557 patients treated at 15 institutions in China between January 2019 and December 2021. Patients were divided into three groups based on chemotherapy regimens: EC-THP (epirubicin, cyclophosphamide, docetaxel/paclitaxel protein-bound, trastuzumab, pertuzumab), TCbHP (docetaxel/paclitaxel protein-bound, carboplatin, trastuzumab, pertuzumab), and THP (docetaxel/paclitaxel protein-bound, trastuzumab, pertuzumab).The primary endpoint was total pathological complete response (tpCR), defined as the absence of invasive disease in both the breast and axillary lymph nodes (ypT0/is, ypN0). Secondary endpoints included axillary pCR (ypN0), breast pCR (ypT0/is), and safety. Propensity score overlap weighting was applied to minimize confounding factors.

**Results:**

Of the 557 patients included in the study, 341 (61.2%) achieved tpCR. The tpCR rate was significantly higher in the HER2-positive hormone receptor (HR)-negative group (183/242 patients, 75.6%) than in the HER2-positive HR-positive group (158/315 patients, 50.2%, *P <* 0.001), as well as bpCR was achieved in 188/241 patients (77.7%) in the HER2-positive HR-negative group compared to 164/315 patients (52.1%) in the HER2-positive HR-positive group (*P* < 0.001), and apCR was achieved in 214/242 patients (88.4%) vs. 240/315 patients (76.2%), respectively (*P* < 0.001). After applying propensity score overlap weighting, no significant differences were observed among the three treatment regimen groups in tpCR (68.4% vs. 63.0% vs. 54.5%, *P* = 0.116), bpCR (71.3% vs. 64.0% vs. 59.6%, *P* = 0.198), or apCR (80.2% vs. 84.4% vs. 73.9%, *P* = 0.173). Gene analysis suggested favorable tpCR trends in patients with TP53, ERBB2, MYC, or CCND1 alterations, though not statistically significant. Most adverse events were grades 1–2, with anemia (254/557, 45.6%), leukopenia (147/557, 26.4%), and reduced ejection fraction (99/421, 23.4%) being the most common. No fatal toxicities were reported.

**Conclusions:**

Trastuzumab and pertuzumab combined with different chemotherapy regimens demonstrated high tpCR rates and manageable safety in HER2-positive breast cancer, particularly in HER2-positive HR-negative patients. The study supports the real-world efficacy of dual HER2-targeted neoadjuvant therapy, though further validation of biomarkers and long-term outcomes is needed.

**HIGHLIGHTS**

- The pathological complete response (pCR) rate in the human epidermal growth factor receptor 2-positive (HER2+) hormone receptor-negative (HR−) group was higher than that in the HER2+ HR+ group.
- After propensity score via overlap weight, there remains no significant difference among the EC-THP, TCbHP, and THP chemotherapy regimens in total pathological complete response (tpCR), breast pCR, and axillary pCR.
- Gene mutation/amplification analysis illustrated that patients with TP53, ERBB2, MYC, or CCND1 were favored by tpCR, but no significant difference.
- The adverse events of double-target combined chemotherapy are controllable clinically.

## Introduction

Breast cancer (BC) represents a significant global public health challenge, ranking as one of the leading causes of cancer-related deaths in women^[1]^. In 2022 alone, an estimated 2.30 million new cases were diagnosed worldwide^[2,3]^. The burden is especially pronounced in China, where BC is not only the most frequent malignancy in women but also exhibits a rising incidence of 3%–4% per year^[4]^. Although advancements in treatment have led to high 5-year survival rates, particularly for early-stage and non-metastatic disease, the sheer volume of new cases and the persistent threat of progression in aggressive subtypes like human epidermal growth factor receptor 2 (HER2)-positive BC underscore its continued impact^[5]^.

HER2-positive BC is an aggressive subtype associated with poor prognosis, accounting for about 20% of cases^[6]^. Neoadjuvant therapy, which aims to achieve pathological complete response (pCR) – a key surrogate for long-term survival – is a standard of care for these patients^[7]^. The development of anti-HER2 targeted therapies, particularly the dual blockade with trastuzumab and pertuzumab, has revolutionized treatment and significantly improved pCR rates^[8,9]^. Consequently, HP-based combination therapy is now recommended by major clinical guidelines for patients with high-risk HER2-positive early BC.

The recommendation for this dual HER2-targeted therapy is supported by robust evidence from pivotal international trials^[10–12]^. For instance, the landmark NeoSphere study demonstrated that the combination of pertuzumab, trastuzumab, and docetaxel resulted in a total pathological complete response (tpCR) rate of 45.8%, which was significantly higher than the 29.0% achieved with trastuzumab and docetaxel alone. In terms of safety^[13]^, the TRYPHAENA trial evaluated the cardiac safety of this combination and reported a low incidence of symptomatic left ventricular systolic dysfunction^[14]^. The most common grade ≥3 adverse events in these trials were neutropenia, leukopenia, and diarrhea, all of which were considered manageable. These foundational studies established the superior efficacy and acceptable safety profile of neoadjuvant dual blockade in Western populations, setting the stage for its global adoption. Furthermore, the PEONY trial confirmed in an Asian population with HER2-positive BC that dual HER2-targeted therapy achieved a tpCR rate of 39.3%, compared to 21.8% in the control group, and was associated with significant long-term survival benefits and an acceptable safety profile^[15–18]^. This established efficacy has paved the way for recent studies focused on optimizing the chemotherapy backbone, often through de-escalation strategies. For instance, the HELEN-006 phase 3 RCT, conducted specifically in a Chinese population, demonstrated that combining trastuzumab and pertuzumab with single-agent nab-paclitaxel resulted in a higher tpCR rate (66.3%) and better tolerability compared to the combination with docetaxel and carboplatin (57.6%)^[19]^. In a similar vein, the neoCARHP study, reported at the 2025 ASCO Annual Meeting, showed that a regimen of a taxane with trastuzumab and pertuzumab provided non-inferior pCR rates and improved tolerability compared to the same regimen with the addition of carboplatin^[20]^.

Although existing real-world studies have demonstrated the efficacy of neoadjuvant therapy combining trastuzumab and pertuzumab with chemotherapy, significant knowledge gaps persist. Specifically, there is a scarcity of direct, head-to-head comparisons between different chemotherapy backbones, and prior studies have often been limited by small sample sizes^[21–32]^. This highlights a critical need to identify the optimal chemotherapy regimen and to explore predictive biomarkers for pCR in a real-world setting. Therefore, this large, multicenter study was conducted to address these gaps by evaluating the comparative efficacy and safety of different neoadjuvant chemotherapy regimens combined with dual HER2 blockade in a Chinese population of patients with HER2-positive BC.

## Methods

### Study design

**Figure 1.**
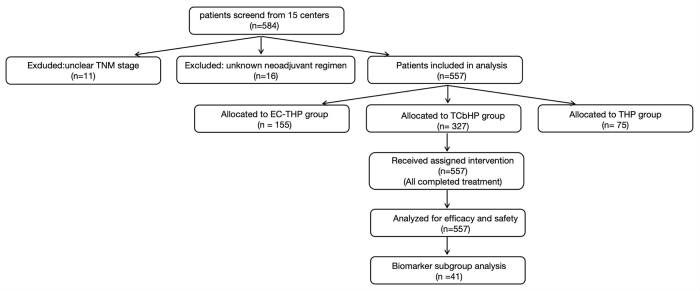
Patient enrollment and analysis flowchart.

This multicenter, retrospective study included 557 patients who were treated at 15 institutions across China between January 2019 and December 2021 (Fig. 1). Before the start of neoadjuvant therapy, all patients were diagnosed by ultrasound-guided biopsy, hematology, and imaging examinations, and distant metastasis was excluded. All participants had an adequate performance status (e.g. ECOG score of 0–1) and sufficient organ function to be considered eligible for neoadjuvant chemotherapy. Eligible patients were divided into three treatment groups according to clinicians’ and patients’ choice: group A received EC-THP treatment (*n* = 155); group B received TCbHP (*n* = 327); and group C received THP treatment (*n* = 75). Breast surgery was performed within 10 weeks after the completion of neoadjuvant therapy. This study was carried out according to the Declaration of Helsinki and was permitted by the Ethics Committee of each joining hospital (Ethics Committee Clinical Trial Approval Number: (B) KY2022170) and Clinical Study Registry (ChiCTR2500102649). The requirement for individual patient informed consent was waived by the Ethics Committee due to the retrospective nature of the study. This study has been reported according to STROCSS criteria and STROCSS guidelines^[33]^.

TNM, tumor, node, metastasis; E, epirubicin; C, cyclophosphamide; T, taxane (e.g. docetaxel, paclitaxel); H, trastuzumab; P, pertuzumab; Cb, carboplatin.

### Participants

Inclusion criteria: women aged 18–80 years; breast masses and/or axillary lymph node biopsies were pathologically confirmed as invasive cancer, and IHC was HER-2 positive [HER-2 (3+) or HER-2 (2+) fluorescence *in situ* hybridization (+)]; early or locally advanced BC; good compliance and receiving targeted therapy of trastuzumab combined with pertuzumab as neoadjuvant chemotherapy. Exclusion criteria: incomplete medical records; intolerant to chemotherapy; neoadjuvant treatment discontinuation for any reason. In this study, genomic profiling was not a routine component of care. It was performed at the discretion of the treating physician and the patient, a decision often influenced by the high cost of the test. The biomarker analysis subgroup (*n* = 41), therefore, comprises a convenience sample of all patients at Southwest Hospital who underwent this optional genomic testing during their diagnostic workup. The potential for selection bias resulting from this non-random sampling is a limitation of this sub-analysis and is addressed in the Discussion section.

### Treatment

All neoadjuvant regimens were administered based on the NCCN guidelines and the Chinese Anti-Cancer Association guidelines. Eligible patients were assigned to one of the following three treatment groups: EC-THP, TCbHP, or THP. The abbreviations represent the following chemotherapy and targeted agents: E (epirubicin), C (cyclophosphamide), T (taxane, either docetaxel or paclitaxel protein-bound), Cb (carboplatin), H (trastuzumab), and P (pertuzumab).

EC-THP Group: Patients first received four 21-day cycles of epirubicin (90–100 mg/m^2^, D1) and cyclophosphamide (600 mg/m^2^, D1). This was followed by a taxane phase consisting of 12 weekly cycles of paclitaxel (80 mg/m^2^, D1). Concurrent with the taxane, dual HER2-targeted therapy was administered, consisting of pertuzumab (loading dose 840 mg, then 420 mg) and trastuzumab (loading dose 8 mg/kg, then 6 mg/kg). Both targeted therapies were given every 21 days for a total of four cycles in the neoadjuvant phase.

TCbHP Group: Patients received six 21-day cycles of treatment. The regimen consisted of a taxane, either docetaxel (75 mg/m^2^, D1) or paclitaxel protein-bound (125 mg/m^2^, D1, D8), combined with carboplatin (AUC = 6, D1), pertuzumab (loading dose 840 mg, then 420 mg, D1), and trastuzumab (loading dose 8 mg/kg, then 6 mg/kg, D1). AUC is the Area under the concentration-time curve, a measure of total drug exposure in the body over a given time.

THP Group: Patients received six 21-day cycles of treatment, with approximately 88% of patients completing all six cycles. The regimen consisted of a taxane, either docetaxel (80–100 mg/m^2^, D1) or paclitaxel protein-bound (125 mg/m^2^, D1, D8), combined with pertuzumab and trastuzumab at the same dosages described above.

Tumor response was assessed every two cycles via breast examination, ultrasound, or MRI, based on the Response Evaluation Criteria in Solid Tumors (RECIST, version 1.1). Patients who experienced disease progression during neoadjuvant therapy were promptly switched to alternative treatments or underwent surgery.

### Efficacy and safety evaluation

The primary endpoint of this study was tpCR. Secondary endpoints included axillary pCR (apCR), breast pCR (bpCR), and safety outcomes.

In this study, tpCR was defined as the absence of invasive carcinoma in both the breast and axillary lymph nodes (ypT0/is, ypN0), allowing for the presence of residual ductal carcinoma *in situ*. ApCR was defined as the absence of invasive carcinoma in the axillary lymph nodes (ypN0), and bpCR was defined as the absence of invasive carcinoma in the breast (ypT0/is).

The Common Terminology Criteria for Adverse Events, version 5.0, was used to evaluate chemotherapy-related adverse events, including anemia, leukopenia, elevated alanine aminotransferase (ALT), and others. Left ventricular ejection fraction (LVEF) was assessed via transthoracic echocardiography at baseline and monitored every 2–3 cycles during neoadjuvant therapy, following institutional standard practice. Cardiotoxicity was defined as a ≥10% decline in LVEF from baseline to below the lower limit of normal (<50%), in accordance with previous studies.

Only patients with available echocardiographic follow-up data were included in the cardiac safety analysis. Due to the retrospective nature of the study, echocardiographic data were inconsistently recorded in routine clinical practice, leading to missing cardiac monitoring data in some cases. The primary endpoint, tpCR, was evaluated at the time of definitive surgery, which was planned to occur within 2–6 weeks following the completion of neoadjuvant therapy. The exact date of surgery was documented for each patient. Adverse events were assessed, recorded, and followed up by the treating physicians during the treatment course.

### Data collection

Data for this study were retrospectively collected from the electronic medical record systems and clinical databases of the 15 participating institutions. Data extraction was independently performed by trained researchers using a standardized data collection form to ensure consistency. The following patient information was obtained: demographic characteristics (e.g. age); ECOG performance status; clinicopathological features (e.g. ER/PR status, T stage, N stage, Ki67 index, HER2 IHC score); neoadjuvant treatment regimens and number of cycles received; and lesion response assessments based on RECIST version 1.1. For a subset of patients, data on gene mutations and amplifications were also collected. To ensure data integrity, all entries were independently verified to minimize transcription errors. All patient data were anonymized prior to analysis to protect patient privacy.

### Statistical analysis

A complete-case analysis approach was adopted for all analyses, and no data imputation was performed for missing values. For the biomarker analysis, only patients with available genomic sequencing data (*n* = 41) were included. Similarly, for the cardiac safety analysis, patients without follow-up echocardiographic assessments (*n* = 136) were excluded.

The discrepancies between the three groups were compared with the Kruskal–Wallis rank sum test for non-normal continuous variables and ordinal categorical variables, and the Pearson χ^2^ test was used for binary categorical variables.

To minimize the effect of confounding factors of real-world data, propensity scores were estimated with multinomial logistic regression, with chemotherapy regimens (EC-THP, TCbHP, and THP) as the outcome and demographic and clinicopathologic characteristics (age, ECOG score, ER, PR, T stage, N stage, Ki67 score, and HER2 IHC score) as pretreatment covariates, followed by overlap weighting to balance the average treatment effect in the overlap population (a population of eligible patients who have no strong preference for the choice of treatments). Compared with other weighting methods, for example, inverse probability weighting, overlap weighting allows for minimizing the influence of extreme propensity scores and achieves a good balance^[34–36]^. The generalized overlap weights of nominal treatments were calculated via a method proposed by Li *et al*^[37]^. To assess balance, standardized mean differences (SMD) of covariates before and after propensity score overlap weighting were calculated^[38]^. SMD <0.1 has been suggested as an adequate balance between groups.

Logistic univariate analysis was used to analyze the correlation between clinicopathological features and pCR rate. Subsequently, variables with a *P*-value <0.1 in the univariate analysis were included in a multivariate logistic regression model to identify independent predictors of tpCR. A significance level of 0.05 for two-sided comparisons was considered statistically significant. All data processing and visualization were performed with R 4.3.2 (https://www.r-project.org/).

Biomarker analysis was performed on a subset of patients with available genomic data. As part of routine clinical practice, pretreatment core biopsy samples were tested in selected patients treated at Southwest Hospital between January 2019 and December 2021. Due to the retrospective nature of the study and the limited reimbursement coverage of gene testing in China, genomic profiling was not universally performed. Only 41 patients had valid genomic data during this period, and biomarker analysis was therefore limited to this subgroup. As this subgroup was not randomly selected, there may be selection bias, and the limited sample size may result in insufficient statistical power to draw definitive conclusions. Therefore, these results should be considered exploratory and serve primarily to generate hypotheses for future research.

## Results

### Patient and treatment characteristics

Between January 2019 and December 2021, a total of 557 patients with HER2-positive early or locally advanced BC were included. The key clinicopathological characteristics of these patients are summarized in Table 1. Among all patients, 182 (32.7%) were younger than 45 years, 242 (43.4%) were aged 45–55 years, and 133 (23.9%) were older than 55 years; the median age was 49 years (interquartile range [IQR], 42–55). A total of 305 patients (54.8%) were premenopausal, and 252 (45.2%) were postmenopausal. HER2-positive HR-positivity was observed in 208 patients (37.3%), while 349 patients (62.7%) were HER2-positive HR-negative. Regarding ECOG performance status, 536 patients (96.2%) had a score of 0, and 21 (3.8%) had a score of 1–2. In terms of clinical stage, 18 patients (3.2%) were stage I, 338 (60.7%) were stage II, and 201 (36.1%) were stage III. As for pathological type, the vast majority of tumors were Invasive carcinoma of no special type (541 patients, 97.1%). Other histological types included Invasive mucinous carcinoma (2 patients, 0.4%) and others (14 patients, 2.5%). Ki67 expression was ≤30% in 266 patients (47.8%) and >30% in 291 patients (52.2%). HER2 immunohistochemistry (IHC) scores were 2+ in 87 patients (15.6%) and 3+ in 470 patients (84.4%). With regard to chemotherapy regimens, 327 patients (58.7%) received TCbHP, 155 (27.8%) received EC-THP, and 75 (13.5%) received THP. The baseline characteristics stratified by HR are listed in Supplemental Digital Content Table 1, available at: http://links.lww.com/JS9/F234. Prior to propensity score weighting, there were significant imbalances in baseline characteristics among the three treatment groups, notably in ECOG PS, ER, PR, and HER2 IHC score (Table 1).

**Table 1:** Clinicopathologic characteristics of enrolled patients, before and after propensity score overlap weighting

		Unweighted					After propensity score overlap weighting				
Characteristics	Total	EC-THP	TCbHP	THP	*P*-value^a^	Standardized mean difference^b^	EC-THP	TCbHP	THP	*P*-value^a^	Standardized mean difference^b^
No. of patients	557	155	327	75			35.3	34.6	34.8		
Age, median [IQR], y	49.0 [42.0, 55.0]	49.0 [42.0, 55.0]	49.0 [41.5, 55.0]	51.0 [44.5, 58.0]	0.122	0.212	50.0 [42.7, 55.0]	50.0 [44.0, 56.0]	48.4 [42.2, 56.0]	0.927	0.025
ECOG PS					0.009	0.175				0.876	0.035
0	536 (96.2)	143 (92.3)	320 (97.9)	73 (97.3)			34.0 (96.3)	33.7 (97.3)	33.6 (96.4)		
1–2	21 (4.2)	12 (7.7)	7 (2.1)	2 (2.7)			1.3 (3.7)	0.9 (2.7)	1.2 (3.6)		
Menopausal state					0.357	0.105					
Premenopausal	305 (54.8)	81 (52.3)	187 (57.2)	37 (49.3)							
Postmenopausal	252 (45.2)	74 (47.7)	140 (42.8)	38 (50.7)							
ER					0.004	0.321				0.921	0.032
Negative	287 (51.5)	67 (43.2)	170 (52.0)	50 (66.7)			20.6 (58.4)	19.8 (57.3)	19.5 (56.0)		
Positive	270 (48.5)	88 (56.8)	157 (48.0)	25 (33.3)			14.7 (41.6)	14.8 (42.7)	15.3 (44.0)		
PR					<0.001	0.445				0.822	0.050
Negative	304 (54.6)	58 (37.4)	194 (59.3)	52 (69.3)			21.1 (59.7)	21.0 (60.7)	19.8 (57.0)		
Positive	253 (45.4)	97 (62.6)	133 (40.7)	23 (30.7)			14.2 (40.3)	13.6 (39.3)	15.0 (43.0)		
HR^c^					\	\					
Negative	242 (43.4)	44 (28.4)	154 (47.1)	44 (58.7)							
Positive	315 (56.6)	111 (71.6)	173 (52.9)	31 (41.3)							
T stage					0.424	0.211				0.917	**0.127**
0–1	47 (8.4)	10 (6.5)	32 (9.8)	5 (6.7)			3.3 (9.4)	2.3 (6.5)	2.0 (5.7)		
2	369 (66.2)	102 (65.8)	216 (66.1)	51 (68.0)			22.2 (62.9)	24.2 (69.8)	24.3 (69.7)		
3	94 (16.9)	30 (19.4)	55 (16.8)	9 (12.0)			5.8 (16.4)	5.3 (15.2)	5.5 (15.7)		
4	47 (8.4)	13 (8.4)	24 (7.3)	10 (13.3)			4.0 (11.3)	2.9 (8.4)	3.1 (9.0)		
N stage					0.151	0.345				0.965	0.068
0	141 (25.3)	32 (20.6)	89 (27.2)	20 (26.7)			9.2 (26.1)	9.0 (26.1)	8.0 (23.0)		
1	286 (51.3)	82 (52.9)	158 (48.3)	46 (61.3)			20.1 (57.1)	19.9 (57.5)	21.4 (61.5)		
2	90 (16.2)	34 (21.9)	50 (15.3)	6 (8.0)			4.4 (12.3)	4.0 (11.6)	3.9 (11.2)		
3	40 (7.2)	7 (4.5)	30 (9.2)	3 (4.0)			1.6 (4.5)	1.7 (4.8)	1.5 (4.3)		
Stage^c^					\	\					
1	18 (3.2)	2 (1.3)	13 (4.0)	3 (4.0)							
2	338 (60.7)	90 (58.1)	196 (59.9)	52 (69.3)							
3	201 (36.1)	63 (40.6)	118 (36.1)	20 (26.7)							
Histological tumor type											
Invasive carcinoma of no special type	541 (97.1)	149 (96.1)	323 (98.8)	69 (92.0)	0.009						
Invasive mucinous carcinoma	2 (0.4)	1 (0.6)	1 (0.3)	0 (0.0)							
Other	14 (2.5)	5 (3.2)	3 (0.9)	6 (8.0)							
Ki67					0.770	0.062				0.940	0.027
≤30	266 (47.8)	74 (47.7)	159 (48.6)	33 (44.0)			15.7 (44.4)	15.9 (45.9)	16.2 (46.4)		
>30	291 (52.2)	81 (52.3)	168 (51.4)	42 (56.0)			19.6 (55.6)	18.7 (54.1)	18.7 (53.6)		
HER2 IHC score					<0.001	0.428				0.706	0.058
2+	87 (15.6)	47 (30.3)	35 (10.7)	5 (6.7)			3.2 (9.1)	4.0 (11.6)	4.1 (11.7)		
3+	470 (84.4)	108 (69.7)	292 (89.3)	70 (93.3)			32.1 (90.9)	30.6 (88.4)	30.7 (88.3)		
Breast operation mode^c^					\	\					
Breast-conserving Surgery	82 (14.7)	17 (11.0)	61 (18.7)	4 (5.3)							
Adenomammectomy	452 (81.1)	131 (84.5)	254 (77.7)	67 (89.3)							
Adenomammectomy + reconstruction	23 (4.0)	7 (4.5)	12 (3.7)	4 (5.3)							
LN dissection method^c^					\	\					
SLN	105 (18.9)	31 (20.0)	64 (19.6)	10 (13.3)							
Axillary dissection	451 (81.0)	123 (79.4)	263 (80.4)	65 (86.7)							
Not done	1 (0.2)	1 (0.6)	0 (0.0)	0 (0.0)							

Data were presented as n (%) unless otherwise specified.

ECOG PS, Eastern Cooperative-Oncology Group performance status; ER, estrogen receptor; PR, progesterone receptor; HR, hormone receptor; HER2, human epidermal growth factor receptor 2; IHC, immunohistochemistry; SLN, sentinel lymph node; LN, lymph node; E, epirubicin; C, cyclophosphamide; T, taxane (docetaxel or paclitaxel protein-bound); Cb, carboplatin; H, trastuzumab; P, pertuzumab.

^a^ Differences in non-normal continuous variables (e.g. age) and ordinal variables (e.g. stage) were evaluated using the Kruskal–Wallis test. Differences in binary categorical variables were calculated with the Pearson χ^2^ test.

^b^ The standardized mean difference (SMD) for all pairwise comparisons was calculated via a method proposed by Flury and Riedwyl. An SMD <0.1 has been suggested as indicating adequate balance between groups.

^c^ HR and Stage were not included in covariates that were used to calculate the propensity score, due to their multicollinearity with other variables; breast operation mode and LN dissection method were not included because they were not baseline characteristics.

### The pCR rate before overlap weighting

**Figure 2.**
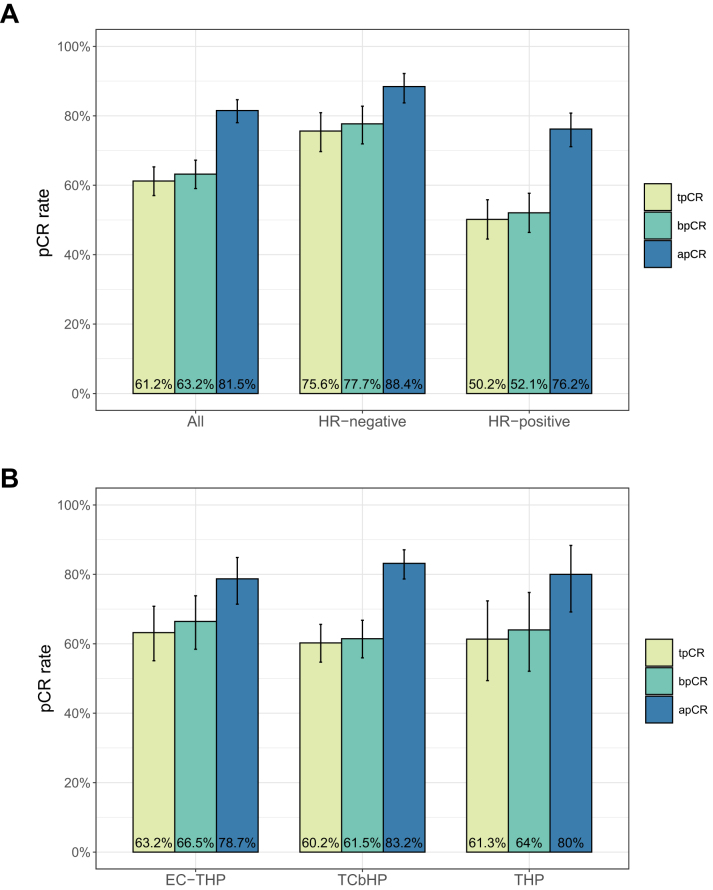
Pathological complete response rates before propensity score weighting. (A) Comparison by HR status. (B) Comparison by neoadjuvant chemotherapy regimen.

As shown in Figure 2A, among all 557 patients, 341 patients (61.2%) achieved tpCR, 352 patients (63.2%) achieved bpCR, and 454 patients (81.5%) achieved apCR.

pCR, pathological complete response; tpCR, total pathological complete response; bpCR, breast pathological complete response; apCR, axillary pathological complete response; HR, hormone receptor; E, epirubicin; C, cyclophosphamide; T, taxane (e.g. docetaxel, paclitaxel); H, trastuzumab; P, pertuzumab; Cb, carboplatin.

The tpCR rate was significantly higher in the HER2-positive HR-negative group (183/242 patients, 75.6%) than in the HER2-positive HR-positive group (158/315 patients, 50.2%, *P* < 0.001). Similarly, the bpCR rate was higher in the HR-negative group compared to the HR-positive group (188/241 patients, 77.7% vs. 164/315 patients, 52.1%, *P* < 0.001), as was the apCR rate (214/242 patients, 88.4% vs. 240/315 patients, 76.2%, *P* < 0.001; Table 2).

**Table 2:** Pathological complete response rates stratified by hormone receptor (HR) status

Variables	Overall	Negative (*n* = 242)	Positive (*n* = 315)	*P*-value
tpCR	341 (61.2)	183 (75.6)	158 (50.2)	<0.001
apCR	454 (81.5)	214 (88.4)	240 (76.2)	<0.001
bpCR	352 (63.2)	188 (77.7)	164 (52.1)	<0.001

Data were presented as *n* (%).

tpCR, total pathological complete response (ypT0/Tis, ypN0); bpCR, breast pathological complete response (ypT0/Tis); apCR, axillary pathological complete response (ypN0).

^a^ *P* values among the two groups were calculated using Pearson’s χ^2^ test. The Pearson’s χ^2^ test was performed using R 4.3.2 (https://www.r-project.org/).

As shown in Figure 2B, among the three chemotherapy regimen groups, tpCR was achieved in 98/155 patients (63.2%) in the EC-THP group, 197/327 patients (60.2%) in the TCbHP group, and 46/75 patients (61.3%) in the THP group. No significant differences in tpCR, bpCR, or apCR rates were observed among the chemotherapy regimens (*P* = 0.821, 0.466, and 0.564, respectively; Table 3).

**Table 3:** Pathological complete response rates among different neoadjuvant chemotherapy regimens, before and after propensity score overlap weighting

		Unweighted				After propensity score overlap weighting			
Variables	Overall	EC-THP (n = 155)	TCbHP (*n* = 327)	THP (*n* = 75)	*P*-value^a^	EC-THP (*n* = 35.3)^b^	TCbHP (*n* = 34.6)^b^	THP (*n* = 34.8)^b^	*P*-value^a^
tpCR	341 (61.2)	98 (63.2)	197 (60.2)	46 (61.3)	0.821	24.2 (68.4)	21.8 (63.0)	19.0 (54.5)	0.116
bpCR	352 (63.2)	122 (78.7)	272 (83.2)	60 (80.0)	0.466	25.2 (71.3)	22.2 (64.0)	20.8 (59.6)	0.198
apCR	454 (81.5)	103 (66.5)	201 (61.5)	48 (64.0)	0.564	28.3 (80.2)	29.2 (84.4)	25.7 (73.9)	0.173

Data were presented as *n* (%).

tpCR, total pathological complete response (ypT0/Tis, ypN0); bpCR, breast pathological complete response (ypT0/Tis); apCR, axillary pathological complete response (ypN0); E, epirubicin; C, cyclophosphamide; T, taxane (docetaxel or paclitaxel protein-bound); Cb, carboplatin; H, trastuzumab; P, pertuzumab.

^a^ *P* values among the three groups were calculated using Pearson’s χ^2^ test.

^b^ Virtual sample size after overlap weighting.

### The pCR rate after overlap weighting

**Figure 3.**
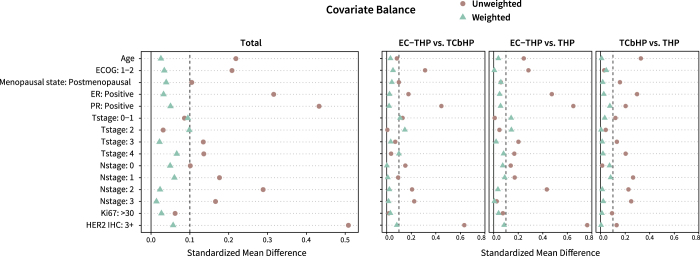
Standardized mean differences of baseline characteristics before and after propensity score overlap weighting.

All 557 eligible patients were included in the propensity score analysis. After overlap weighting, the baseline characteristics, including age, ECOG score, ER, PR, T stage, N stage, Ki67 score, and HER2 IHC score, were well-balanced across the three treatment groups (Table 1, Fig. 3). As shown in Table 3, no significant differences in the rates of tpCR (*P =* 0.116), bpCR (*P =* 0.198), or apCR (*P =* 0.173) were observed among the three chemotherapy regimens after weighting.

E, epirubicin; C, cyclophosphamide; T, taxane (e.g. docetaxel, paclitaxel); H, trastuzumab; P, pertuzumab; Cb, carboplatin.

### Predictors of pathological complete response

**Figure 4.**
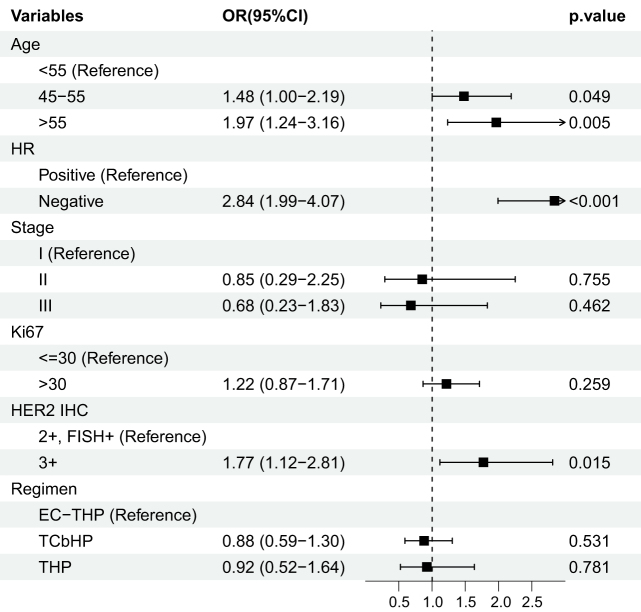
Forest plot of univariate analysis for predictors of tpCR.

OR, odds ratio; CI, confidence interval; HR, hormone receptor; HER2, human epidermal growth factor receptor 2; IHC, immunohistochemistry; FISH, fluorescence *in situ* hybridization; E, epirubicin; C, cyclophosphamide; T, taxane; H, trastuzumab; P, pertuzumab; Cb, carboplatin; tpCR, total pathological complete response.

Univariate logistic regression analysis was performed to identify factors associated with achieving tpCR. The results suggested that younger age (<55 years), HR-negative status, and a HER2 IHC score of 3+ were significantly associated with a higher likelihood of tpCR (all *P <* 0.05; Fig. 4). Subsequently, these variables were included in a multivariate logistic regression model. The analysis confirmed that HR-negative status was a significant independent predictor of achieving tpCR (odds ratio [OR], 2.80; 95% CI, 1.92–4.11; *P* < 0.001). However, younger age and a HER2 IHC score of 3+ were no longer statistically significant in the multivariate analysis (Supplemental Digital Content Table 2, available at: http://links.lww.com/JS9/F235).

### Relationship between gene alterations and the tpCR

**Figure 5.**
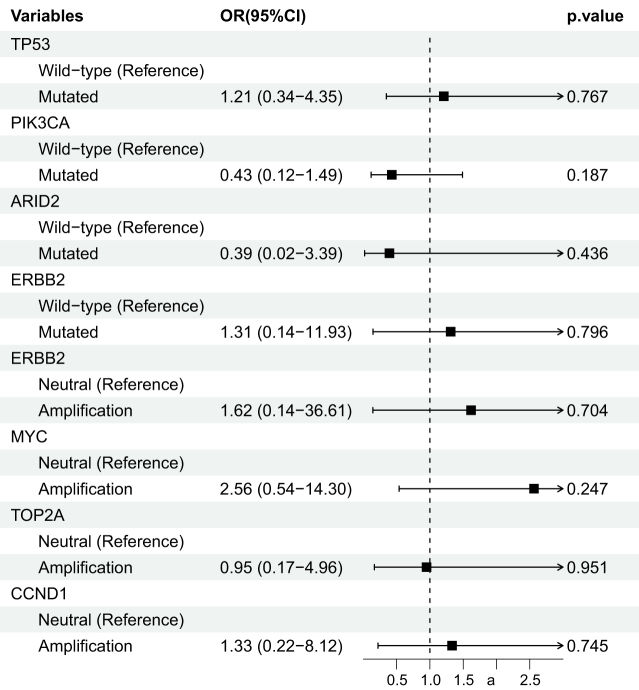
Forest plot of univariate analysis for the association between key gene alterations and tpCR.

OR, odds ratio; CI, confidence interval; TP53, tumor protein p53; PIK3CA, phosphatidylinositol-4,5-bisphosphate 3-kinase catalytic subunit alpha; ARID2, AT-rich interaction domain 2; ERBB2, erb-b2 receptor tyrosine kinase 2; MYC, MYC proto-oncogene; TOP2A, DNA topoisomerase II alpha; CCND1, cyclin D1; tpCR, total pathological complete response.

We used univariate analysis to explore the relationship between gene alterations and the tpCR in a subset of 41 patients. The baseline characteristics of this biomarker subgroup are detailed in Table 4. Among these 41 patients, TP53, PIK3CA, ERBB2, and MYC mutation/amplification were the most commonly observed genetic alterations (Table 5). Gene mutation/amplification analysis demonstrated that patients with TP53, ERBB2, MYC, or CCND1 alterations showed a trend toward higher tpCR rates (OR >1) compared to patients without these alterations (Fig. 5), though these differences did not reach statistical significance, likely due to the limited sample size. As this analysis included only 41 patients (accounting for 7.4% of the total cohort), the statistical power was limited, and no adjustment for multiple comparisons was performed. Therefore, the results should be interpreted with caution as exploratory findings.

**Table 4:** Baseline characteristics of the biomarker subgroup (*n* = 41)

Characteristics	Level	Overall
N		41
Age (median [IQR])		47 (44, 53)
Age group (%)	<45	13 (31.7)
	45–55	23 (56.1)
	>55	5 (12.2)
ECOG score (%)	0	41 (100.0)
ER (%)	Negative	23 (56.1)
	Positive	18 (43.9)
PR (%)	Negative	27 (65.9)
	Positive	14 (34.1)
HR (%)	Negative	22 (53.7)
	Positive	19 (46.3)
Menopausal status (%)	Premenopausal	22 (53.7)
	Postmenopausal	19 (46.3)
T stage (%)	0	0 (0.0)
	1	7 (17.1)
	2	27 (65.9)
	3	4 (9.8)
	4	3 (7.3)
N stage (%)	0	10 (24.4)
	1	27 (65.9)
	2	1 (2.4)
	3	3 (7.3)
Stage (%)	1	6 (14.6)
	2	26 (63.4)
	3	9 (22.0)
Histological type (%)	Invasive carcinoma of no special type	41 (100.0)
Ki67 (%)	≤30	27 (65.9)
	>30	14 (34.1)
HER2 IHC (%)	2+	1 (2.4)
	3+	40 (97.6)
Preoperative chemotherapy regimen (%)	EC-THP	3 (7.3)
	TCbHP	36 (87.8)
	THP	2 (4.9)

Data were presented as *n* (%).

IQR, interquartile range; ECOG, Eastern Cooperative Oncology Group; ER, estrogen receptor; PR, progesterone receptor; HR, hormone receptor; HER2, human epidermal growth factor receptor 2; IHC, immunohistochemistry; Ki67, Ki-67 proliferation index; E, epirubicin; C, cyclophosphamide; T, taxane (docetaxel or paclitaxel protein-bound); Cb, carboplatin; H, trastuzumab; P, pertuzumab.

**Table 5:** Gene mutation/amplification frequency (*n* = 41)

Mutant genes	N	Rate	Amplified genes	N	Rate
TP53	24	0.585	ERBB2	38	0.927
PIK3CA	23	0.561	MYC	8	0.195
ARID2	4	0.098	TOP2A	7	0.171
ERBB2	4	0.098	CCND1	6	0.146
ATRX	3	0.073	ZNF217	3	0.073
MAP3K1	3	0.073	CCNE1	2	0.049
MLH3	3	0.073	FGF19	2	0.049
NRG1	3	0.073	GNAS	2	0.049
NSD1	3	0.073	MDM2	2	0.049
WRN	3	0.073	AURKB	1	0.024

### Safety evaluation of neoadjuvant therapy

Among all 557 patients who completed neoadjuvant therapy, the most common adverse reactions were anemia, occurring in 254 patients (45.6% for all grades) with 20 patients (3.6%) experiencing grade 3 or higher severity. Leukopenia occurred in 147 patients (26.4% for all grades) with 18 patients (3.2%) experiencing grade 3 or higher severity. ALT elevation was observed in 82 patients (14.7% for all grades) with only 2 patients (0.4%) experiencing grade 3 or higher severity. Febrile neutropenia occurred in 82 patients (14.7% for all grades) with 11 patients (2.0%) experiencing grade 3 or higher severity. Thrombocytopenia was observed in 56 patients (10.1% for all grades) with 6 patients (1.1%) experiencing grade 3 or higher severity. Regarding cardiac toxicity, echocardiographic monitoring data were available for 421 patients (cardiac echocardiographic monitoring data were absent in 136 cases). Reduced ejection fraction occurred in 99 patients (23.4% for all grades) among those with available cardiac monitoring data, with 89 patients (21.1%) experiencing grade 2 toxicity and 9 patients (2.1%) experiencing grade 3 toxicity (Table 6).

**Table 6:** Incidence of adverse reactions

Adverse event	All grade (*n*, %)	Grade 1 (*n*, %)	Grade 2 (*n*, %)	Grade 3 (*n*, %)	Grade 4 (*n*, %)
Anemia	254 (45.6)	188 (33.8)	46 (8.3)	20 (3.6)	0
Leukopenia	147 (26.4)	92 (16.5)	37 (6.6)	15 (2.7)	3 (0.5)
ALT elevation	82 (14.7)	65 (11.7)	15 (2.7)	2 (0.4)	0
Febrile neutropenia	82 (14.7)	51 (9.2)	20 (3.6)	11 (2)	0
Thrombocytopenia	56 (10.1)	36 (6.5)	14 (2.5)	6 (1.1)	0
Decreased ejection fraction^a^	99 (23.4)	NA	89 (21.1)	9 (2.1)	1 (0.2)

NA, no data.

^a^ Absence of cardiac echocardiographic monitoring data in 136 cases.

## Discussion

This large, multicenter, real-world study provides key insights into neoadjuvant therapy for HER2-positive BC in a Chinese population. Our principal findings demonstrate that dual HER2 blockade with trastuzumab and pertuzumab achieves high pCR rates, with comparable efficacy observed across different standard chemotherapy backbones after propensity score adjustment. Furthermore, our results confirm that HR-negative status is a strong independent predictor of achieving pCR, underscoring the biological heterogeneity within HER2-positive disease.

Our findings are consistent with the established efficacy of dual HER2 blockade. Numerous pivotal randomized controlled trials (RCTs) have confirmed that adding pertuzumab to trastuzumab-based chemotherapy significantly improves pCR rates, and key trial results are summarized in this paper (Table 7). Similarly, a growing body of real-world evidence has supported these findings in routine clinical practice, and a summary of relevant studies is also provided (Table 8). The overall total pCR (tpCR) rate in our study (61.2%) aligns well with the outcomes reported in both these landmark RCTs and other real-world studies from Western populations. In addition, the safety profile observed in our study, including the low incidence of grade ≥3 cardiotoxicity (2.1% among assessed patients) and other manageable adverse events, was consistent with international data, further supporting the generalizability of the regimen’s safety across different ethnic populations.

**Table 7:** Summary of the results of randomized controlled trials

Study	Phase	N	Scheme (neoadjuvant – adjuvant)	Neoadjuvant treatment cycle	pCR rate	EFS/DFS		OS	Safety LVSD ≥ grade 3
NeoSphere	II	417	TH-FECH	4	29%	5 y EFS: 81%	5 y DFS:81%	NA	0%
									3%
									0%
									0%
					45.8%				
						vs. 86%	vs. 84%		
			vs. THP-FECH			vs. 73%	vs. 80%		
						vs. 73%	vs. 75%		
					16.8%				
			vs. PH-FECH						
					24%				
			vs. TP-FECH						
PEONY	III	329	PHT-FECPH	4	39.3%	5 y EFS:84.8%	5 y DFS:86%	5 y OS:93%	–
			vs. TH-FECH						
					21.8%	vs. 73.7%	vs. 75%	vs. 90%	
TRYPHAENA	II	225	FEC-T + HP (FECx3-Tx3, HPx6)	6	61.6%	3 y DFS: 87% vs. 88% vs. 90%		3 y OS: 94% vs. 94%	0%
					57.3%				
								vs. 93%	
					66.2%				

			vs. FEC-THP (FECx3-Tx3, HPx3)						
			vs. TCHP (TCbx6, HPx6)						
									3%
									0%
KRISTINE	III	444	TCHP	6	56%	3 y EFS: 94.2% vs. 85.3%	3 y iDFS: 93% vs. 92%	NA	–
					44%				
			vs. KP (K:T-DM1)						
BERENICE	II	397	ddAC-THP	8	61.8%	5 y EFS: 90.8% vs. 89.2%		5 y OS: 96.1% vs. 93.8%	2%
									0%
			vs. FEC-THP						
					60.7%				
TRAIN-2		438	FECx3 + PH-TCbHPx6	9	67%	3 y EFS: 93.5% vs. 92.7%		3 y OS: 98.% vs. 97.7%	1%
									0%
					68%				
			vs. TCbHPx9						
HELEN-006	III	689	nab-PHP	6	66.3%	NA		NA	–
			vs. TCbHP						
					57.6				
neoCARHP	III	774	THP	6	64.1%	NA		NA	–
			vs. TCbHP						
					65.9%				

pCR, pathological complete response; EFS, event-free survival; DFS, disease-free survival; OS, overall survival; LVSD, left ventricular systolic dysfunction; NA, not available or not reported; dd, dose-dense; FEC, a combination of fluorouracil, epirubicin, and cyclophosphamide; AC, a combination of doxorubicin (adriamycin) and cyclophosphamide; H, trastuzumab; P, pertuzumab; T, taxane (e.g. docetaxel, paclitaxel); Cb, carboplatin; nab-P, nab-paclitaxel; K, trastuzumab emtansine (T-DM1).

**Table 8:** Summary of the results of real-world research

No.	Title	Year	Scheme	Country	Journal	Patients	Main results
1[33]	Pathologic complete response with neoadjuvant doxorubicin and cyclophosphamide followed by paclitaxel with trastuzumab and pertuzumab in patients with HER2-positive early stage breast cancer: a single center experience	2017	AC-THP	Germany	*Oncologist*	*N* = 57	tpCR: 72%
2[34]	Pathological outcomes of HER2-positive non-metastatic breast cancer patients treated with neoadjuvant dual anti-HER2 therapy and taxane: an Australian experience	2019	THP	Australia	*Asia Pac J Clin Oncol*	*N* = 19	tpCR: 68%
3[35]	Real-world effectiveness of dual HER2 blockade with pertuzumab and trastuzumab for neoadjuvant treatment of HER2-positive early breast cancer (The NEOPETRA Study)	2020	HP + T	Spain	*Breast Cancer Res Treat*	*N* = 243	tpCR: 66.4%
			HP + E + T				
							HP + E + T scheme tpCR:71%
			HP + Cb				
							HP + T scheme: 59.3%
							HP + platinum scheme tpCR: 48.6%.
4[36]	Neoadjuvant docetaxel with or without carboplatin plus dual HER2 blockade for HER2-positive breast cancer: a retrospective multicenter Chinese study	2020	TCbHP	China	*Gland Surg*	N = 72	tpCR: 70.8%
			THP				
							TCbHP scheme tpCR: 76.1%
							THP scheme tpCR: 61.5%
5[37]	Real-world evidence of neoadjuvant docetaxel/carboplatin/trastuzumab/pertuzumab (TCHP) in patients with HER2-positive early or locally advanced breast cancer: a single-institutional clinical experience	2021	TCHP	Korea	*Cancer Res Treat*	*N* = 447	tpCR:64%
							3 y EFS: 90.6%
6[38]	Pathologic complete response rates after neoadjuvant pertuzumab and trastuzumab with chemotherapy in early stage HER2-positive breast cancer – increasing rates of breast conserving surgery: a real-world experience	2021	EC-THP	Hungary	*Pathol Oncol Res*	*N* = 82	tpCR: 54%
			FEC-THP				
7[39]	Neoadjuvant pertuzumab plus trastuzumab in combination with anthracycline-free chemotherapy regimen in patients with HER2-positive breast cancer-real-world data from a single center in India	2021	TCHP	India	*Cancer Treat Res Commun*	*N* = 45	tpCR: 55.6%
8[40]	Neoadjuvant pertuzumab plus trastuzumab in combination with docetaxel and carboplatin in patients with HER2-positive breast cancer: real-world data from the National Institute of Oncology in Poland	2022	TCbHP	Netherlands	*Cancers (Basel)*	N = 34	tpCR: 52.9%
9[41]	Pertuzumab study in the neoadjuvant setting for HER2-positive nonmetastatic breast cancer in Australia (PeRSIA)	2022	AC/EC-THP; THP-AC/EC	Australia	*Int J Cancer*	*N* = 95	bpCR: 70.7%
							tpCR: 64.1%
			THP				
10[23]	Real-world study of trastuzumab and pertuzumab combined with chemotherapy in neoadjuvant treatment for patients with HER2-positive breast cancer	2022	TcbHP	China	*Medicine*	302	tpCR: 64.9%
							bpCR: 73.5%
			THP				
			AC-THP				
11[22]	Neoadjuvant therapy for early human epidermal growth factor receptor 2-positive breast cancer in China. A multicenter real-world study (CSBrS-015)	2022	TC	China	*Multicenter Study*	1032	tpCR 34.5% vs. 57.9%
			TPC				
12[42]	Efficacy and safety of neoadjuvant pertuzumab plus trastuzumab in combination with chemotherapy regimen in Chinese patients with HER2-positive early breast cancer: a real-world retrospective multicenter cohort study	2022	TCbHP	China	*Cancer Res*	188	pCR: 46.8%
			THP				
			AC-THP				
13	De-escalation of neoadjuvant taxane and carboplatin therapy in HER2-positive breast cancer with dual HER2 blockade: a multicenter real-world experience in China	2024	TCbHP	China	*World J Surg Oncol*	220	tpCR 66% vs. 53%
			THP				

tpCR, total pathological complete response; bpCR, breast pathological complete response; EFS, event-free survival; DFS, disease-free survival; OS, overall survival; AC, a regimen including doxorubicin and cyclophosphamide; EC, a regimen including epirubicin and cyclophosphamide; FEC, a regimen including fluorouracil, epirubicin, and cyclophosphamide; H, trastuzumab; P, pertuzumab; T, taxane (e.g. docetaxel, paclitaxel); Cb, carboplatin.

Compared to other real-world studies from China, our multicenter cohort is one of the largest and includes stratified analyses by both HR status and chemotherapy regimen. The significantly higher tpCR rate among HER2-positive/HR-negative patients (75.6%) vs. HR-positive patients (50.2%) aligns with global evidence and provides robust, specific data for the Chinese population.

A key finding of our study is the lack of a statistically significant difference in tpCR rates among the TCbHP, EC-THP, and THP regimens after propensity score weighting. This suggests that when combined with potent dual HER2 blockade, the choice of chemotherapy backbone may not be the primary driver of efficacy. This finding has significant clinical implications, supporting a more flexible and personalized approach where regimen selection can be tailored based on individual patient profiles, such as toxicity risk (e.g. avoiding anthracyclines in patients with cardiac risk factors), comorbidities, and drug accessibility, without compromising the likelihood of achieving pCR.

As expected, HR status remains a key determinant of response. Our cohort confirmed that tpCR rates were significantly higher in HER2-positive HR-negative patients than in HER2-positive HR-positive counterparts, a finding consistent with both prior RCTs and real-world data. The biological basis for this difference may relate to the complex bidirectional crosstalk between the HER2 and estrogen receptor signaling pathways. On one hand, high HR expression may attenuate the efficacy of anti-HER2 therapy. On the other hand, HER2 overexpression can activate downstream pathways such as MAPK and PI3K-AKT, disrupting ER signaling^[39,40]^. This mutual interference may render single-pathway targeting insufficient, ultimately resulting in lower pCR rates in the HER2-positive HR-positive subgroup. This finding strongly suggests the need for novel strategies in this subgroup, such as the integration of endocrine therapy or CDK4/6 inhibitors into the neoadjuvant regimen.

While our biomarker analysis was exploratory, it aligns with emerging concepts. The trend toward higher tpCR rates in patients with TP53 mutations and lower rates in those with PIK3CA mutations is consistent with previous reports and suggests genomic profiling could help identify patients unlikely to respond to standard therapy^[41–43]^. From a translational perspective, future strategies may rely on a deeper biological understanding. Recent transcriptome-based profiling has identified distinct HER2-positive subtypes (e.g. HER2-CLA, HER2-IM, HER2-BM, HER2-LUM), each with unique therapeutic vulnerabilities^[44]^. Incorporating such molecular classifications into neoadjuvant decision-making, for instance, prioritizing standard regimens for the HER2-CLA subtype while exploring immunotherapy for HER2-IM or CDK4/6 inhibitors for HER2-LUM could enhance treatment precision, moving beyond clinicopathological features alone.

Although numerous studies have been conducted in this field, our study makes a unique contribution to the existing evidence base. We provide one of the largest multicenter, real-world datasets from a Chinese population, comprising 557 patients. In contrast to previous real-world studies, which were often limited by smaller sample sizes or a focus on single regimens, our research not only evaluates efficacy and safety but also performs an adjusted comparison of different chemotherapy backbones. By applying propensity score overlap weighting, our finding of comparable efficacy among regimens offers a crucial evidence-based rationale for individualized treatment selection, addressing a critical data gap for this specific population.

This study has several limitations. First, its retrospective design may introduce selection bias, and the follow-up period is too short to evaluate long-term outcomes like EFS or OS. Second, the biomarker analysis was limited to a small, non-randomly selected subset of 41 patients from a single center, limiting its generalizability and requiring validation in larger, prospective studies. Third, the safety data, while informative, were retrospectively collected and may have overlooked certain adverse events.

In conclusion, this large real-world study provides robust evidence supporting the efficacy and manageable safety of dual HER2-targeted neoadjuvant therapy in Chinese patients with HER2-positive breast cancer. The comparable efficacy across different chemotherapy backbones empowers clinicians with the flexibility to personalize treatment, balancing the high probability of achieving pCR with individual patient toxicity profiles and characteristics.

## Supplementary Material

## Data Availability

The data that support the findings of this study are available from the corresponding author upon reasonable request.

## Research registration unique identifying number (UIN)

The study was registered in the Chinese Clinical Trial Registry (Registration Number: ChiCTR2500102649).
