# Supplementary material for: Efficacy, safety, and biomarkers of neoadjuvant trastuzumab and pertuzumab combined with chemotherapy in Chinese patients with HER2-positive breast cancer: a multicenter retrospective cohort study
Source: Int J Surg. 2025 Sep 30;112(1):1318–31. doi: 10.1097/JS9.0000000000003551 (PMC12825773; doi:10.1097/JS9.0000000000003551)
Supplement: Supplementary file 1 [file js9-112-1318-001.docx]

Supplemental Table 1. Baseline Characteristics of Patients Stratified by Hormone Receptor (HR) Status

| **Characteristics** | **Level** | **Overall** | **Negative** | **Positive** | ***P* value^a^** |
| --- | --- | --- | --- | --- | --- |
| **N** |  | 557 | 242 | 315 |  |
| **Age (median [IQR])** |  | 49.00 (42.00, 55.00) | 51.00 (45.00, 57.00) | 47.00 (41.00, 53.50) | < 0.001 |
| **Age Group** | < 45 | 182 (32.7) | 60 (24.8) | 122 (38.7) | < 0.001 |
|  | 45-55 | 242 (43.4) | 105 (43.4) | 137 (43.5) |  |
|  | > 55 | 133 (23.9) | 77 (31.8) | 56 (17.8) |  |
| **ECOG score** | 0 | 536 (96.2) | 231 (95.5) | 305 (96.8) | 0.390 |
|  | 1 | 20 (3.6) | 11 (4.5) | 9 (2.9) |  |
|  | 2 | 1 (0.2) | 0 (0.0) | 1 (0.3) |  |
| **ER** | negative | 287 (51.5) | 242 (100.0) | 45 (14.3) | < 0.001 |
|  | positive | 270 (48.5) | 0 (0.0) | 270 (85.7) |  |
| **PR** | negative | 304 (54.6) | 242 (100.0) | 62 (19.7) | < 0.001 |
|  | positive | 253 (45.4) | 0 (0.0) | 253 (80.3) |  |
| **HR** | negative | 242 (43.4) | 242 (100.0) | 0 (0.0) | < 0.001 |
|  | positive | 315 (56.6) | 0 (0.0) | 315 (100.0) |  |
| **Menopausal status** | Premenopausal | 305 (54.8) | 111 (45.9) | 194 (61.6) | < 0.001 |
|  | Postmenopausal | 252 (45.2) | 131 (54.1) | 121 (38.4) |  |
| **T stage** | 0 | 2 (0.4) | 1 (0.4) | 1 (0.3) | 0.233 |
|  | 1 | 45 (8.1) | 25 (10.3) | 20 (6.3) |  |
|  | 2 | 369 (66.2) | 164 (67.8) | 205 (65.1) |  |
|  | 3 | 94 (16.9) | 36 (14.9) | 58 (18.4) |  |
|  | 4 | 47 (8.4) | 16 (6.6) | 31 (9.8) |  |
| **N stage** | 0 | 141 (25.3) | 58 (24.0) | 83 (26.3) | 0.017 |
|  | 1 | 286 (51.3) | 126 (52.1) | 160 (50.8) |  |
|  | 2 | 90 (16.2) | 32 (13.2) | 58 (18.4) |  |
|  | 3 | 40 (7.2) | 26 (10.7) | 14 (4.4) |  |
| **Stage** | 1 | 18 (3.2) | 9 (3.7) | 9 (2.9) | 0.743 |
|  | 2 | 338 (60.7) | 149 (61.6) | 189 (60.0) |  |
|  | 3 | 201 (36.1) | 84 (34.7) | 117 (37.1) |  |
| **Histological type** | Invasive carcinoma of no special type | 541 (97.1) | 235 (97.1) | 306 (97.1) | 0.410 |
|  | Invasive mucinous carcinoma | 2 (0.4) | 0 (0.0) | 2 (0.6) |  |
|  | Other | 14 (2.5) | 7 (2.9) | 7 (2.2) |  |
| **Ki67** | < = 30 | 266 (47.8) | 114 (47.1) | 152 (48.3) | 0.855 |
|  | > 30 | 291 (52.2) | 128 (52.9) | 163 (51.7) |  |
| **HER2 IHC** | 2 + | 87 (15.6) | 16 (6.6) | 71 (22.5) | < 0.001 |
|  | 3 + | 470 (84.4) | 226 (93.4) | 244 (77.5) |  |
| **chemotherapy regimen** | EC-THP | 155 (27.8) | 44 (18.2) | 111 (35.2) | < 0.001 |
|  | TCbHP | 327 (58.7) | 154 (63.6) | 173 (54.9) |  |
|  | THP | 75 (13.5) | 44 (18.2) | 31 (9.8) |  |
| **Breast operation mode** | Breast-conserving | 82 (14.7) | 35 (14.5) | 47 (14.9) | 0.022 |
|  | Adenomammectomy | 452 (81.1) | 203 (83.9) | 249 (79.0) |  |
|  | Adenomammectomy + reconstruction | 23 (4.1) | 4 (1.6) | 19 (6.0) |  |
| **LN dissection method** | SLN | 105 (18.9) | 58 (24.0) | 47 (14.9) | 0.013 |
|  | Not done | 1 (0.2) | 1 (0.4) | 0 (0.0) |  |
|  | Axillary dissection | 451 (81.0) | 183 (75.6) | 268 (85.1) |  |

Data were presented as n (%) unless otherwise specified.

Abbreviations: ECOG PS, Eastern Cooperative Oncology Group performance status; ER, Estrogen receptor; PR, Progesterone receptor; HR, Hormone receptor; HER2, human epidermal growth factor receptor 2; IHC, Immunohistochemistry; T, Taxane (Docetel or Paclitaxel protein-bound); Cb, Carboplatin; H, Trastuzumab; P, Pertuzumab.

^a^ Differences between groups were evaluated using the Kruskal-Wallis Rank Sum Test for non-normal continuous variables and ordinal categorical variables, and the Pearson χ² Test for binary categorical variables.
