# Supplementary material for: Efficacy, safety, and biomarkers of neoadjuvant trastuzumab and pertuzumab combined with chemotherapy in Chinese patients with HER2-positive breast cancer: a multicenter retrospective cohort study
Source: Int J Surg. 2025 Sep 30;112(1):1318–31. doi: 10.1097/JS9.0000000000003551 (PMC12825773; doi:10.1097/JS9.0000000000003551)
Supplement: Supplementary file 2 [file js9-112-1318-002.docx]

Supplemental Table 2. Multivariate Analysis of Predictors for tpCR

| **Variables** | **Term** | **OR(95%CI)** | ***p.*value** | **OR** | **LL** | **UL** |
| --- | --- | --- | --- | --- | --- | --- |
| **Age** | <45 |  |  |  |  |  |
|  | 45-55 | 1.34 (0.89-2.01) | 0.157 | 1.339 | 0.893 | 2.008 |
|  | >55 | 1.55 (0.95-2.54) | 0.078 | 1.553 | 0.954 | 2.544 |
| **HR** | Positive |  |  |  |  |  |
|  | Negative | 2.80 (1.92-4.11) | <0.001 | 2.801 | 1.924 | 4.113 |
| **HER2 IHC** | 2+ |  |  |  |  |  |
|  | 3+ | 1.31 (0.81-2.12) | 0.278 | 1.306 | 0.805 | 2.116 |
